# 3-(2,4-Dichloro­phen­yl)-1,5-di-2-furylpentane-1,5-dione

**DOI:** 10.1107/S1600536809001548

**Published:** 2009-01-17

**Authors:** Hoong-Kun Fun, Reza Kia, P. S. Patil, S. M. Dharmaprakash

**Affiliations:** a X-ray Crystallography Unit, School of Physics, Universiti Sains Malaysia, 11800 USM, Penang, Malaysia; b Department of Studies in Physics, Mangalore University, Mangalagangotri, Mangalore 574 199, India

## Abstract

In the title compound, C_19_H_14_Cl_2_O_4_, intra­molecular C—H⋯O and C—H⋯Cl hydrogen bonds generate *S*(6) and *S*(5) ring motifs, respectively. In the crystal structure, inter­molecular C—H⋯O inter­actions between symmetry-related mol­ecules involving two methyl­ene groups and an O atom as a bifurcated acceptor generate an *R*_2_^1^(6) ring motif. In the mol­ecule, one of the furan rings is rotationally disordered by approximately 180° about the single C—C bond to which it is attached; the refined site-occupancy factors are 0.505 (7) and 0.495 (7). The major component of the disordered furan ring and the benzene ring form a dihedral angle of 88.8 (4)°. The dihedral angle between the major disorder component and the other furan ring is 81.9 (4)°. In addition, the crystal structure is stabilized by further inter­molecular C—H⋯O (×2) hydrogen bonds and C—H⋯π inter­actions.

## Related literature

For details of hydrogen-bond motifs, see: Bernstein *et al.* (1995 ▶). For related structures and physico-chemical properties, see, for example: Li *et al.* (2004 ▶); Patil, Teh *et al.* (2007 ▶); Patil, Fun *et al.* (2007 ▶). For bond-length data, see: Allen *et al.* (1987 ▶).
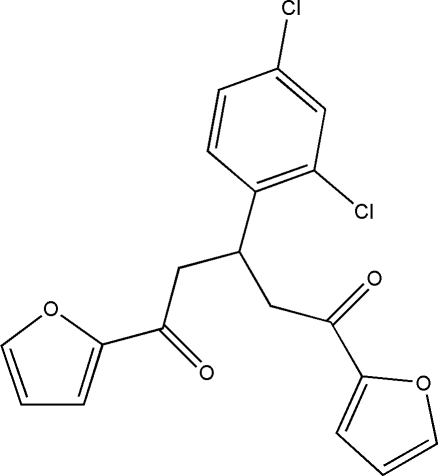


## Experimental

### 

#### Crystal data

C_19_H_14_Cl_2_O_4_
*M* *_r_* = 377.20
Monoclinic, 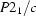

*a* = 9.9116 (1) Å
*b* = 17.7480 (3) Å
*c* = 10.1173 (2) Å
β = 107.612 (1)°
*V* = 1696.32 (5) Å^3^
*Z* = 4
Mo *K*α radiation
μ = 0.40 mm^−1^
*T* = 100.0 (1) K
0.22 × 0.14 × 0.05 mm

#### Data collection

Bruker SMART APEXII CCD area-detector diffractometer
Absorption correction: multi-scan (**SADABS**; Bruker, 2005 ▶) *T*_min_ = 0.918, *T*_max_ = 0.981
29237 measured reflections
6622 independent reflections
5037 reflections with *I* > 2˘*I*)
*R*_int_ = 0.042

#### Refinement

*R*[*F* ^2^ > 2σ(*F* ^2^)] = 0.041
*wR*(*F* ^2^) = 0.113
*S* = 1.10
6622 reflections
263 parameters
H-atom parameters constrained
Δρ_max_ = 0.47 e Å^−3^
Δρ_min_ = −0.36 e Å^−3^

### 

Data collection: *APEX2* (Bruker, 2005 ▶); cell refinement: *SAINT* (Bruker, 2005 ▶); data reduction: *SAINT*; program(s) used to solve structure: *SHELXTL* (Sheldrick, 2008 ▶); program(s) used to refine structure: *SHELXTL*; molecular graphics: *SHELXTL*; software used to prepare material for publication: *SHELXTL* and *PLATON* (Spek, 2003 ▶).

## Supplementary Material

Crystal structure: contains datablocks global, I. DOI: 10.1107/S1600536809001548/lh2756sup1.cif

Structure factors: contains datablocks I. DOI: 10.1107/S1600536809001548/lh2756Isup2.hkl

Additional supplementary materials:  crystallographic information; 3D view; checkCIF report

## Figures and Tables

**Table 1:** Hydrogen-bond geometry (Å, °)

*D*—H⋯*A*	*D*—H	H⋯*A*	*D*⋯*A*	*D*—H⋯*A*
C6—H6*A*⋯O3	0.97	2.53	3.1213 (16)	119
C6—H6*B*⋯O2^i^	0.97	2.49	3.3194 (15)	143
C7—H7*A*⋯Cl1	0.98	2.57	3.0739 (12)	112
C8—H8*B*⋯O2^i^	0.97	2.56	3.4492 (15)	152
C1—H1*A*⋯*Cg*1^ii^	0.93	2.97	3.6095 (16)	127

Symmetry codes: (i) 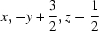; (ii) 
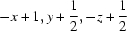
. *Cg*1 is the centroid of the C14–C19 benzene ring.

**Fig. 1.**
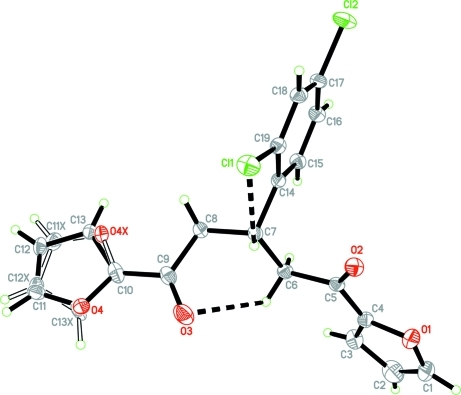
The molecular structure of the title compound, showing 50% probability displacement ellipsoids and the atomic numbering. Open bonds indicate the minor disordered component. Dashed lines show intramolecular hydrogen bonds.

**Fig. 2.**
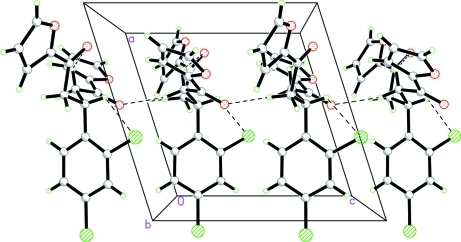
The crystal packing of the major component of (I), viewed down the b-axis, showing a chain of molecules along the c-axis. Intramolecular and intermolecular interactions are drawn as dashed lines.

## supplementary crystallographic information

### Comment

Over the past several decades, linear π-conjugated organic molecules have attracted considerable interest because of their promising applications (such as for organic light-emitting diodes, non-linear optical properties, conductivity and photocells) due to their delocalized π systems (Li *et al.*, 2004; Patil, Teh *et al.*, 2007; Patil, Fun *et al.*, 2007). In the course of our synthesis of the π-conjugated organic molecule, the title compound, (Fig. 1) was synthesized and its crystal structure is reported here.

In the title molecular structure (Fig.1), one of the furane rings has an approximately 180° rotational disorder (atoms of the minor part are lablled with the suffix *X*) about the C9—C10 single bond. The bond lengths (Allen *et al.*, 1987) and angles are within the normal values. The ratio of the refined site-occupancy factors of the major and minor parts of the disordered furane ring is 0.505 (7)/0.495 (7). The major part of the disordered furane ring and the benzene ring are twisted from each other by the dihedral angle of 88.8 (4)°. Intramolecular C—H···O and C—H···Cl hydrogen bonds (Table 1), generate *S*(6) and *S*(5) ring motifs, respectively. Intermolecular C—H···O interactions (Table 1) between the neighbouring molecules involving two methylene groups and an oxygen atom as a bifurcated acceptor generate *R*_2_^1^(6) ring motif. In the crystal packing (Fig. 2), intermolecular C—H···O interactions link neighbouring molcules into a chain along the *c* axis.

### Experimental

The title compound was synthesized by the condensation of 2,4-Dichlorobenzaldehyde (0.01 mol, 1.75 mg) with 2-acetylfuran (0.02 mol, 2.02 ml) in methanol (80 ml) in the presence of a catalytic amount of sodium hydroxide solution (5 ml, 30%). After stirring (6 h), the contents of the flask were poured into ice-cold water (500 ml) and left to stand for 5 h. The resulting crude solid was filtered and dried. Crystals suitable for *X*-ray analysis were grown by slow evaporation of an acetone solution at room temperature.

### Refinement

All hydrogen atoms were postioned geometrically in the riding model approximation with C—H= 0.93 Å, and *U*_iso_ (H) = 1.2*U*_eq_ (C). Initially rigid, similarity and simulation restraints were applied to the disordered furane ring. After steady state has been reached, these restraints were removed for the final refinement. No restraint was used in the final refinement.

### Crystal data

**Table:**

C_19_H_14_Cl_2_O_4_	*F*(000) = 776
*M**_r_* = 377.20	*D*_x_ = 1.477 Mg m^−^^3^
Monoclinic, *P*2_1_/*c*	Mo *K*α radiation, λ = 0.71073 Å
Hall symbol: -P 2ybc	Cell parameters from 6655 reflections
*a* = 9.9116 (1) Å	θ = 2.4–33.5°
*b* = 17.7480 (3) Å	µ = 0.40 mm^−^^1^
*c* = 10.1173 (2) Å	*T* = 100 K
β = 107.612 (1)°	Block, colourless
*V* = 1696.32 (5) Å^3^	0.22 × 0.14 × 0.05 mm
*Z* = 4	

### Data collection

**Table:**

Bruker SMART APEXII CCD area-detector diffractometer	6622 independent reflections
Radiation source: fine-focus sealed tube	5037 reflections with *I* > 2˘*I*)
graphite	*R*_int_ = 0.042
φ and ω scans	θ_max_ = 33.6°, θ_min_ = 2.3°
Absorption correction: multi-scan (*SADABS*; Bruker, 2005)	*h* = −15→15
*T*_min_ = 0.918, *T*_max_ = 0.981	*k* = −27→27
29237 measured reflections	*l* = −15→14

### Refinement

**Table:**

Refinement on *F*^2^	Primary atom site location: structure-invariant direct methods
Least-squares matrix: full	Secondary atom site location: difference Fourier map
*R*[*F*^2^ > 2σ(*F*^2^)] = 0.041	Hydrogen site location: inferred from neighbouring sites
*wR*(*F*^2^) = 0.113	H-atom parameters constrained
*S* = 1.10	*w* = 1/[σ^2^(*F*_o_^2^) + (0.0518*P*)^2^ + 0.2913*P*] where *P* = (*F*_o_^2^ + 2*F*_c_^2^)/3
6622 reflections	(Δ/σ)_max_ = 0.001
263 parameters	Δρ_max_ = 0.47 e Å^−^^3^
0 restraints	Δρ_min_ = −0.36 e Å^−^^3^

### Special details

**Table:**

Experimental. The low-temperature data was collected with the Oxford Cyrosystem Cobra low-temperature attachment.
Geometry. All e.s.d.'s (except the e.s.d. in the dihedral angle between two l.s. planes) are estimated using the full covariance matrix. The cell e.s.d.'s are taken into account individually in the estimation of e.s.d.'s in distances, angles and torsion angles; correlations between e.s.d.'s in cell parameters are only used when they are defined by crystal symmetry. An approximate (isotropic) treatment of cell e.s.d.'s is used for estimating e.s.d.'s involving l.s. planes.
Refinement. Refinement of *F*^2^ against ALL reflections. The weighted *R*-factor *wR* and goodness of fit *S* are based on *F*^2^, conventional *R*-factors *R* are based on *F*, with *F* set to zero for negative *F*^2^. The threshold expression of *F*^2^ > σ(*F*^2^) is used only for calculating *R*-factors(gt) *etc*. and is not relevant to the choice of reflections for refinement. *R*-factors based on *F*^2^ are statistically about twice as large as those based on *F*, and *R*- factors based on ALL data will be even larger.

### Fractional atomic coordinates and isotropic or equivalent isotropic displacement parameters (Å^2^)

**Table:**

	*x*	*y*	*z*	*U*_iso_*/*U*_eq_	Occ. (<1)
Cl1	0.38051 (3)	0.605962 (19)	0.52165 (3)	0.02599 (8)	
Cl2	−0.09927 (3)	0.69351 (2)	0.14183 (4)	0.03110 (9)	
O1	0.67813 (10)	0.93790 (6)	0.42430 (11)	0.0302 (2)	
O2	0.53954 (9)	0.80850 (5)	0.46030 (9)	0.02198 (18)	
O3	0.81229 (10)	0.60583 (6)	0.43590 (11)	0.0304 (2)	
C1	0.75581 (16)	0.98987 (8)	0.3790 (2)	0.0407 (4)	
H1A	0.7774	1.0378	0.4164	0.049*	
C2	0.79639 (17)	0.96291 (9)	0.2745 (2)	0.0405 (4)	
H2A	0.8500	0.9879	0.2271	0.049*	
C3	0.74192 (14)	0.88814 (8)	0.24929 (15)	0.0254 (3)	
H3A	0.7525	0.8548	0.1822	0.031*	
C4	0.67168 (12)	0.87570 (7)	0.34297 (13)	0.0194 (2)	
C5	0.60146 (11)	0.80821 (6)	0.37231 (12)	0.0168 (2)	
C6	0.61549 (12)	0.73848 (6)	0.29237 (13)	0.0179 (2)	
H6A	0.7151	0.7268	0.3108	0.021*	
H6B	0.5763	0.7486	0.1939	0.021*	
C7	0.54043 (12)	0.66983 (6)	0.32935 (13)	0.0171 (2)	
H7A	0.5721	0.6647	0.4305	0.021*	
C8	0.58102 (12)	0.59720 (6)	0.26816 (13)	0.0198 (2)	
H8A	0.5156	0.5576	0.2739	0.024*	
H8B	0.5708	0.6054	0.1708	0.024*	
C9	0.73035 (13)	0.57107 (7)	0.34030 (13)	0.0214 (2)	
C10	0.77185 (14)	0.49951 (7)	0.29298 (14)	0.0242 (3)	
O4	0.8896 (11)	0.4658 (4)	0.3532 (8)	0.0238 (17)	0.505 (7)
C11	0.8971 (13)	0.3977 (7)	0.3023 (9)	0.0210 (13)	0.505 (7)
H11A	0.9737	0.3652	0.3341	0.025*	0.505 (7)
C12	0.7754 (9)	0.3813 (4)	0.1953 (9)	0.0206 (10)	0.505 (7)
H12A	0.7551	0.3376	0.1423	0.025*	0.505 (7)
C13	0.6897 (8)	0.4453 (3)	0.1847 (8)	0.0176 (9)	0.505 (7)
H13A	0.5998	0.4527	0.1228	0.021*	0.505 (7)
O4X	0.6852 (6)	0.4628 (3)	0.2027 (6)	0.0189 (8)	0.495 (7)
C11X	0.7518 (9)	0.3984 (4)	0.1882 (10)	0.0251 (13)	0.495 (7)
H11B	0.7088	0.3603	0.1269	0.030*	0.495 (7)
C12X	0.8834 (13)	0.3948 (7)	0.2692 (10)	0.0250 (17)	0.495 (7)
H12B	0.9482	0.3563	0.2735	0.030*	0.495 (7)
C13X	0.9064 (19)	0.4644 (7)	0.3512 (14)	0.0261 (17)	0.495 (7)
H13B	0.9856	0.4809	0.4210	0.031*	0.495 (7)
C14	0.38037 (12)	0.67715 (6)	0.28323 (12)	0.0166 (2)	
C15	0.30627 (12)	0.70752 (7)	0.15451 (13)	0.0192 (2)	
H15A	0.3569	0.7252	0.0970	0.023*	
C16	0.15938 (12)	0.71228 (7)	0.10920 (13)	0.0211 (2)	
H16A	0.1125	0.7328	0.0229	0.025*	
C17	0.08406 (12)	0.68587 (7)	0.19492 (14)	0.0209 (2)	
C18	0.15200 (12)	0.65386 (7)	0.32252 (13)	0.0199 (2)	
H18A	0.1008	0.6358	0.3792	0.024*	
C19	0.29875 (12)	0.64942 (6)	0.36363 (12)	0.0175 (2)	

### Atomic displacement parameters (Å^2^)

**Table:**

	*U*^11^	*U*^22^	*U*^33^	*U*^12^	*U*^13^	*U*^23^
Cl1	0.02556 (15)	0.03169 (16)	0.02196 (16)	−0.00052 (11)	0.00903 (12)	0.00834 (11)
Cl2	0.01501 (13)	0.0454 (2)	0.03221 (19)	−0.00001 (12)	0.00608 (12)	−0.00328 (14)
O1	0.0295 (5)	0.0250 (5)	0.0360 (6)	−0.0014 (4)	0.0100 (4)	−0.0086 (4)
O2	0.0226 (4)	0.0261 (4)	0.0190 (4)	0.0000 (3)	0.0089 (3)	−0.0021 (3)
O3	0.0255 (5)	0.0352 (5)	0.0257 (5)	0.0090 (4)	0.0007 (4)	0.0001 (4)
C1	0.0310 (7)	0.0182 (6)	0.0712 (12)	−0.0024 (5)	0.0129 (7)	−0.0048 (7)
C2	0.0357 (8)	0.0300 (7)	0.0614 (11)	0.0016 (6)	0.0229 (8)	0.0172 (7)
C3	0.0285 (6)	0.0256 (6)	0.0246 (6)	0.0034 (5)	0.0116 (5)	0.0034 (5)
C4	0.0194 (5)	0.0191 (5)	0.0177 (5)	0.0004 (4)	0.0029 (4)	−0.0009 (4)
C5	0.0140 (4)	0.0203 (5)	0.0146 (5)	0.0006 (4)	0.0021 (4)	−0.0007 (4)
C6	0.0168 (5)	0.0198 (5)	0.0184 (5)	−0.0010 (4)	0.0073 (4)	−0.0017 (4)
C7	0.0160 (5)	0.0175 (5)	0.0186 (5)	0.0007 (4)	0.0063 (4)	0.0010 (4)
C8	0.0174 (5)	0.0183 (5)	0.0242 (6)	0.0018 (4)	0.0068 (4)	0.0006 (4)
C9	0.0216 (5)	0.0226 (5)	0.0218 (6)	0.0047 (4)	0.0094 (4)	0.0058 (4)
C10	0.0263 (6)	0.0244 (6)	0.0261 (6)	0.0093 (5)	0.0144 (5)	0.0089 (5)
O4	0.020 (3)	0.0189 (18)	0.027 (2)	−0.0004 (16)	−0.0008 (16)	−0.0070 (17)
C11	0.0140 (17)	0.025 (2)	0.023 (3)	−0.0002 (14)	0.003 (2)	−0.003 (2)
C12	0.028 (2)	0.013 (2)	0.0247 (17)	0.0017 (17)	0.0136 (17)	−0.0035 (18)
C13	0.0187 (15)	0.014 (2)	0.0185 (19)	−0.0006 (19)	0.0035 (12)	−0.0019 (15)
O4X	0.0193 (10)	0.014 (2)	0.0229 (18)	0.0024 (14)	0.0063 (11)	−0.0018 (12)
C11X	0.029 (3)	0.019 (3)	0.031 (2)	−0.003 (2)	0.014 (2)	−0.007 (2)
C12X	0.024 (3)	0.0161 (15)	0.036 (5)	0.0023 (18)	0.010 (3)	−0.008 (3)
C13X	0.016 (2)	0.024 (3)	0.035 (3)	0.0092 (16)	0.0019 (18)	0.012 (3)
C14	0.0165 (5)	0.0154 (5)	0.0192 (5)	−0.0006 (4)	0.0074 (4)	−0.0011 (4)
C15	0.0191 (5)	0.0205 (5)	0.0194 (6)	0.0003 (4)	0.0080 (4)	0.0010 (4)
C16	0.0189 (5)	0.0235 (5)	0.0203 (6)	0.0012 (4)	0.0051 (4)	0.0001 (4)
C17	0.0145 (5)	0.0230 (5)	0.0254 (6)	−0.0010 (4)	0.0062 (4)	−0.0055 (4)
C18	0.0194 (5)	0.0210 (5)	0.0222 (6)	−0.0033 (4)	0.0105 (4)	−0.0032 (4)
C19	0.0191 (5)	0.0168 (5)	0.0172 (5)	−0.0005 (4)	0.0065 (4)	−0.0004 (4)

### Geometric parameters (Å, °)

**Table:**

Cl1—C19	1.7388 (12)	C10—O4	1.289 (10)
Cl2—C17	1.7373 (12)	C10—C13X	1.427 (16)
O1—C1	1.3663 (19)	C10—C13	1.499 (7)
O1—C4	1.3668 (15)	O4—C11	1.324 (14)
O2—C5	1.2247 (14)	C11—C12	1.386 (13)
O3—C9	1.2249 (16)	C11—H11A	0.9300
C1—C2	1.329 (3)	C12—C13	1.403 (9)
C1—H1A	0.9300	C12—H12A	0.9300
C2—C3	1.426 (2)	C13—H13A	0.9300
C2—H2A	0.9300	O4X—C11X	1.350 (7)
C3—C4	1.3531 (18)	C11X—C12X	1.315 (14)
C3—H3A	0.9300	C11X—H11B	0.9300
C4—C5	1.4603 (16)	C12X—C13X	1.466 (18)
C5—C6	1.5077 (16)	C12X—H12B	0.9300
C6—C7	1.5318 (16)	C13X—H13B	0.9300
C6—H6A	0.9700	C14—C15	1.3954 (17)
C6—H6B	0.9700	C14—C19	1.3988 (16)
C7—C14	1.5177 (15)	C15—C16	1.3904 (16)
C7—C8	1.5354 (16)	C15—H15A	0.9300
C7—H7A	0.9800	C16—C17	1.3859 (17)
C8—C9	1.5116 (16)	C16—H16A	0.9300
C8—H8A	0.9700	C17—C18	1.3845 (18)
C8—H8B	0.9700	C18—C19	1.3888 (16)
C9—C10	1.4596 (18)	C18—H18A	0.9300
C10—O4X	1.234 (5)		
			
C1—O1—C4	105.77 (12)	O4—C10—C13	105.1 (4)
C2—C1—O1	111.13 (13)	C13X—C10—C13	104.7 (6)
C2—C1—H1A	124.4	C9—C10—C13	130.8 (3)
O1—C1—H1A	124.4	C10—O4—C11	112.9 (8)
C1—C2—C3	106.75 (13)	O4—C11—C12	110.9 (10)
C1—C2—H2A	126.6	O4—C11—H11A	124.5
C3—C2—H2A	126.6	C12—C11—H11A	124.5
C4—C3—C2	105.87 (13)	C11—C12—C13	104.7 (8)
C4—C3—H3A	127.1	C11—C12—H12A	127.7
C2—C3—H3A	127.1	C13—C12—H12A	127.7
C3—C4—O1	110.47 (11)	C12—C13—C10	106.3 (6)
C3—C4—C5	130.85 (12)	C12—C13—H13A	126.8
O1—C4—C5	118.59 (11)	C10—C13—H13A	126.8
O2—C5—C4	121.26 (11)	C10—O4X—C11X	105.8 (6)
O2—C5—C6	122.77 (10)	C12X—C11X—O4X	113.5 (8)
C4—C5—C6	115.93 (10)	C12X—C11X—H11B	123.2
C5—C6—C7	113.04 (9)	O4X—C11X—H11B	123.2
C5—C6—H6A	109.0	C11X—C12X—C13X	105.4 (11)
C7—C6—H6A	109.0	C11X—C12X—H12B	127.3
C5—C6—H6B	109.0	C13X—C12X—H12B	127.3
C7—C6—H6B	109.0	C10—C13X—C12X	99.7 (10)
H6A—C6—H6B	107.8	C10—C13X—H13B	130.1
C14—C7—C6	113.36 (9)	C12X—C13X—H13B	130.1
C14—C7—C8	108.96 (9)	C15—C14—C19	116.42 (10)
C6—C7—C8	111.30 (9)	C15—C14—C7	121.74 (10)
C14—C7—H7A	107.7	C19—C14—C7	121.72 (10)
C6—C7—H7A	107.7	C16—C15—C14	122.28 (11)
C8—C7—H7A	107.7	C16—C15—H15A	118.9
C9—C8—C7	113.62 (10)	C14—C15—H15A	118.9
C9—C8—H8A	108.8	C17—C16—C15	118.80 (12)
C7—C8—H8A	108.8	C17—C16—H16A	120.6
C9—C8—H8B	108.8	C15—C16—H16A	120.6
C7—C8—H8B	108.8	C18—C17—C16	121.34 (11)
H8A—C8—H8B	107.7	C18—C17—Cl2	119.24 (9)
O3—C9—C10	120.79 (11)	C16—C17—Cl2	119.42 (10)
O3—C9—C8	123.03 (11)	C17—C18—C19	118.19 (11)
C10—C9—C8	116.14 (11)	C17—C18—H18A	120.9
O4X—C10—O4	115.7 (4)	C19—C18—H18A	120.9
O4X—C10—C13X	115.4 (6)	C18—C19—C14	122.92 (11)
O4X—C10—C9	120.0 (3)	C18—C19—Cl1	117.07 (9)
O4—C10—C9	123.6 (4)	C14—C19—Cl1	120.01 (9)
C13X—C10—C9	124.4 (6)		
			
C4—O1—C1—C2	0.25 (18)	C11—C12—C13—C10	0.3 (9)
O1—C1—C2—C3	−0.04 (19)	O4X—C10—C13—C12	−166 (3)
C1—C2—C3—C4	−0.19 (17)	O4—C10—C13—C12	0.1 (7)
C2—C3—C4—O1	0.35 (15)	C13X—C10—C13—C12	4.8 (9)
C2—C3—C4—C5	−175.99 (13)	C9—C10—C13—C12	−172.2 (4)
C1—O1—C4—C3	−0.37 (15)	O4—C10—O4X—C11X	−5.6 (8)
C1—O1—C4—C5	176.48 (12)	C13X—C10—O4X—C11X	−0.6 (9)
C3—C4—C5—O2	−178.14 (13)	C9—C10—O4X—C11X	−176.2 (4)
O1—C4—C5—O2	5.76 (17)	C13—C10—O4X—C11X	9(3)
C3—C4—C5—C6	4.29 (19)	C10—O4X—C11X—C12X	−1.0 (10)
O1—C4—C5—C6	−171.80 (10)	O4X—C11X—C12X—C13X	2.0 (14)
O2—C5—C6—C7	1.96 (16)	O4X—C10—C13X—C12X	1.6 (12)
C4—C5—C6—C7	179.49 (10)	O4—C10—C13X—C12X	96 (12)
C5—C6—C7—C14	67.88 (13)	C9—C10—C13X—C12X	177.1 (6)
C5—C6—C7—C8	−168.86 (10)	C13—C10—C13X—C12X	−0.2 (11)
C14—C7—C8—C9	−162.04 (10)	C11X—C12X—C13X—C10	−2.0 (13)
C6—C7—C8—C9	72.23 (13)	C6—C7—C14—C15	41.31 (15)
C7—C8—C9—O3	−1.98 (17)	C8—C7—C14—C15	−83.23 (13)
C7—C8—C9—C10	175.93 (10)	C6—C7—C14—C19	−142.87 (11)
O3—C9—C10—O4X	175.4 (3)	C8—C7—C14—C19	92.60 (13)
C8—C9—C10—O4X	−2.6 (4)	C19—C14—C15—C16	1.82 (17)
O3—C9—C10—O4	5.5 (6)	C7—C14—C15—C16	177.86 (11)
C8—C9—C10—O4	−172.5 (6)	C14—C15—C16—C17	−0.04 (18)
O3—C9—C10—C13X	0.1 (8)	C15—C16—C17—C18	−1.23 (18)
C8—C9—C10—C13X	−177.8 (7)	C15—C16—C17—Cl2	178.40 (9)
O3—C9—C10—C13	176.6 (4)	C16—C17—C18—C19	0.61 (18)
C8—C9—C10—C13	−1.4 (5)	Cl2—C17—C18—C19	−179.03 (9)
O4X—C10—O4—C11	2.2 (11)	C17—C18—C19—C14	1.33 (17)
C13X—C10—O4—C11	−86 (12)	C17—C18—C19—Cl1	−177.82 (9)
C9—C10—O4—C11	172.5 (7)	C15—C14—C19—C18	−2.49 (17)
C13—C10—O4—C11	−0.6 (10)	C7—C14—C19—C18	−178.53 (11)
C10—O4—C11—C12	0.8 (14)	C15—C14—C19—Cl1	176.63 (9)
O4—C11—C12—C13	−0.7 (12)	C7—C14—C19—Cl1	0.59 (15)

### Hydrogen-bond geometry (Å, °)

**Table:**

*D*—H···*A*	*D*—H	H···*A*	*D*···*A*	*D*—H···*A*
C6—H6A···O3	0.97	2.53	3.1213 (16)	119
C6—H6B···O2^i^	0.97	2.49	3.3194 (15)	143
C7—H7A···Cl1	0.98	2.57	3.0739 (12)	112
C8—H8B···O2^i^	0.97	2.56	3.4492 (15)	152
C1—H1A···Cg1^ii^	0.93	2.97	3.6095 (16)	127

Symmetry codes: (i) *x*, −*y*+3/2, *z*−1/2; (ii) −*x*+1, *y*+1/2, −*z*+1/2.
